# DNA repair genes *RAD52* and *SRS2*, a cell wall synthesis regulator gene *SMI1*, and the membrane sterol synthesis scaffold gene *ERG28* are important in efficient *Agrobacterium*-mediated yeast transformation with chromosomal T-DNA

**DOI:** 10.1186/s12866-016-0672-0

**Published:** 2016-04-02

**Authors:** Yuta Ohmine, Yukari Satoh, Kazuya Kiyokawa, Shinji Yamamoto, Kazuki Moriguchi, Katsunori Suzuki

**Affiliations:** Department of Biological Science, Graduate School of Science, Hiroshima University, Kagamiyama 1-3-1, Higashi-Hiroshima, Hiroshima 739-8526 Japan

**Keywords:** *Agrobacterium*-mediated transformation, Artificial chromosome, DNA repair, Cell surface interaction, Horizontal gene transfer, Yeast, *Saccharomyces cerevisiae*, T-DNA, Trans-domain gene transfer

## Abstract

**Background:**

Plant pathogenic *Agrobacterium* strains can transfer T-DNA regions of their Ti plasmids to a broad range of eukaryotic hosts, including fungi, *in vitro*. In the recent decade, the yeast *Saccharomyces cerevisiae* is used as a model host to reveal important host proteins for the *Agrobacterium*-mediated transformation (AMT). Further investigation is required to understand the fundamental mechanism of AMT, including interaction at the cell surface, to expand the host range, and to develop new tools. In this study, we screened a yeast mutant library for low AMT mutant strains by advantage of a chromosome type T-DNA, which transfer is efficient and independent on integration into host chromosome.

**Results:**

By the mutant screening, we identified four mutant strains (*srs2Δ*, *rad52Δ*, *smi1Δ* and *erg28Δ*), which showed considerably low AMT efficiency. Structural analysis of T-DNA product replicons in AMT colonies of mutants lacking each of the two DNA repair genes, *SRS2* and *RAD52*, suggested that the genes act soon after T-DNA entry for modification of the chromosomal T-DNA to stably maintain them as linear replicons and to circularize certain T-DNA simultaneously. The cell wall synthesis regulator *SMI1* might have a role in the cell surface interaction between the donor and recipient cells, but the *smi1Δ* mutant exhibited pleiotropic effect, i.e. low effector protein transport as well as low AMT for the chromosomal T-DNA, but relatively high AMT for integrative T-DNAs. The ergosterol synthesis regulator/enzyme-scaffold gene *ERG28* probably contributes by sensing a congested environment, because growth of *erg28Δ* strain was unaffected by the presence of donor bacterial cells, while the growth of the wild-type and other mutant yeast strains was suppressed by their presence.

**Conclusions:**

*RAD52* and the DNA helicase/anti-recombinase gene *SRS2* are necessary to form and maintain artificial chromosomes through the AMT of chromosomal T-DNA. A sterol synthesis scaffold gene *ERG28* is important in the high-efficiency AMT, possibly by avoiding congestion. The involvement of the cell wall synthesis regulator *SMI1* remains to be elucidated.

## Background

*Agrobacterium tumefaciens* causes crown gall disease on dicotyledonous plants by delivering a transfer DNA (T-DNA) region derived from its tumor-inducing (Ti) plasmid [1] into plant cells at the infected site. Bacterial factors essential for the T-DNA transfer process have been studied in detail. The Ti plasmid encodes a set of virulence (*vir*) genes. A relaxase protein, VirD2, is one of these *vir* gene product proteins. The protein makes a nick at two 25-base direct repeat border sequences (RB and LB) that define the T-DNA region, and releases single-stranded T-DNA from the Ti plasmid. VirD2 remains covalently attached to the 5′ end of the single-stranded T-DNA and the complex is transported into plant cells through a type IV secretion system (T4SS) channel comprising mainly of proteins encoded by the *virB* operon. In parallel with the T-DNA, effector proteins, such as single-stranded DNA binding protein VirE2, are also mobilized into plant cells [2, 3]. VirE2 binds to the T-DNA in the plant cytoplasm and is thought to protect the T-DNA against nucleases [4]**,** and also ensures nuclear targeting of the complex by virtue of its nuclear localization signal [5]. After entry into the nucleus, T-DNA is integrated dominantly into the nuclear genome in a process of DNA repair via non-homologous end-joining (NHEJ) [6]. Alternatively, the DNA repair machinery also mediates formation of complex extrachromosomal T-DNA structures including circular T-DNA (T-circle) molecules [7].

Recently, to study the T-DNA transfer processes after transport into host cells, host factors involved in the processes have been characterized extensively using the crucifer plant *Arabidopsis thaliana* and the yeast *Saccharomyces cerevisiae*. The host plant protein VirE2-interacting protein 1 (VIP1) binds to VirE2 and acts as a molecular adaptor between VirE2 and a nuclear import machinery importin α to help direct VirE2 to the host nucleus [8, 9], although VIP1 seems dispensable in *Agrobacterium*-mediated transformation (AMT) [10]. Van Attikum et al*.* [11, 12] investigated the recipient factors using yeast. Their studies revealed that T-DNA integration into the recipient genome requires the DNA repair pathway of either NHEJ or homologous recombination (HR). The linear T-DNA is a substrate for integration into the recipient genome, while it is also a substrate for ligation between T-DNA molecules or itself in plant and yeast cells [13]. They also demonstrated that the formation of circularized structures involves the HR pathway in yeast. Genome-wide screens using a set of yeast nonessential gene deletion collections showed that chromatin modification by histone acetyltransferases and deacetylases affects AMT strongly [14]. However, it remains unknown how host yeast genes contribute to the T-DNA transfer process. Compared with the donor bacterial factors, the host factors are not well characterized. One such factor is a component of cell surface structures that must be recognized by the T4SS machinery.

In this study, we sought additional host factors for AMT. The donor *Agrobacterium* strain used in this screen has an autonomous replicable T-DNA that contains a yeast artificial chromosome (YAC) DNA. The T-DNA does not require integration into the recipient yeast genome, and it enables not only high efficiency AMT [15, 16], but also minimizes co-cultivation time to 1 day, compared with the several days of incubation that are necessary for the integration type T-DNA [11, 12]. Consequently, we identified four mutant strains with dramatically decreased AMT efficiencies. Two of these strains lack genes involved in DNA repair and the remaining were mutated for genes that are essential to arrange cell surface structures. Our results indicated that the DNA repair genes are important to maintain the chromosomal type T-DNA in the yeast cells, and that the integrity of the cell surface structures is required for high-efficiency AMT.

## Results

### Identification of yeast chromosomal genes affecting AMT

To find additional host factors that affect AMT, we screened a set of yeast mutant strains for mutants defective in the ability to be transformed by AMT. The donor *Agrobacterium* strain EHA105 was equipped with the binary plasmid pBY1 [16]. pBY1 contains a YAC in the T-DNA region. The T-DNA in pBY1 consists of the selectable markers *URA3* and *TRP1*, autonomous replication and segregation factors *ARS1/CEN4* and two telomere sequences. Therefor the T-DNA is not required to integrate into yeast genome and can greatly elevate AMT compared with integrative type T-DNA [16]. In an initial screening, yeast strains incubated with the donor strain on AB induction agar formed confluent Ura^+^ colonies on SC-ura agar and the proportions of transformant colonies of each strain were confirmed visually. Among the yeast knockout strains in the collection screened, 199 mutant strains showed apparently fewer Ura^+^ colonies compared with the wild-type strain. Previously, Soltani et al. [14] screened yeast genes involved in AMT using yeast mutant collections and identified some genes. In our screening, the genes that they identified did not remain within the selected 199 strains. Subsequently, we evaluated the mutants based on AMT efficiency, which is defined as the ratio of Ura^+^ cell number per output recipient cell number, because a large number of yeast mutants are hypersensitive to stresses and tend to die [17]. We selected mutant strains which showed an AMT efficiency less than a quarter of the wild-type efficiency. As shown in Table 1, four mutant strains, namely, *srs2Δ*, *rad52Δ*, *smi1Δ* and *erg28Δ*, exhibited about 5–24.5 % of the efficiency of the wild-type strain (1.9 × 10^−3^). Srs2 and Rad52 are involved in double-strand break (DSB) repair [18, 19], Erg28 is important for ergosterol synthesis [20, 21], and Smi1 is responsible for regulation of cell wall synthesis [22]. Complementation tests certified that the reductions in AMT efficiencies were caused by the deletion of each gene. The mutant strains with the corresponding wild-type gene on a plasmid pRS313 recovered the AMT efficiencies to levels comparable to the wild-type strain transformed with the empty vector (Table 1).

**Table 1:** Yeast mutant strains exhibiting low AMT efficiency

Yeast strain (genotype)	AMT efficiency^a^	AMT efficiency of complemented strain^b^	TKC efficiency^c^	Growth of yeast cells (fold)^d^		Growth ratio
	% of wild type ± SD	% of wild type ± SD	% of wild type ± SD	(A) with donor cells	(B) w/o donor cells	(A/B)
wild type	(100)	(100)	(100)	3.5 ± 1.7	11.8 ± 4.1	0.30
*srs2Δ*	5.6 ± 2.8**	103.2 ± 21.7	102.2 ± 22.2	5.3 ± 2.2	15.3 ± 6.9	0.34
*rad52Δ*	4.8 ± 1.9**	187.9 ± 41.1*	52.5 ± 27.2*	4.7 ± 0.2	6.1 ± 0.6	0.77
*smi1Δ*	5.0 ± 2.4**	87.8 ± 5.1*	49.8 ± 27.2*	4.8 ± 2.2	10.0 ± 3.8	0.48
*erg28Δ*	24.5 ± 9.5**	123.3 ± 70.4	25.1 ± 18.6**	8.4 ± 1.5	8.2 ± 1.3	1.02

^a^ Yeast strains were cocultivated with *Agrobacterium* strain EHA105 (pBY1). The AMT efficiency of the wild type yeast strain was (1.9 ± 0.1) × 10^-3^

^b^ Each mutant strain was introduced a corresponding wild type gene cloned in centromeric vector pRS313 (see Table 4). The AMT efficiency of the wild type strain harboring the pRS313 vector was (4.1 ± 1.7) × 10^-3^

^c^ Yeast strains were cocultivated with *E. coli* strain HB101 (pRH210, pAY205) for trans-kingdom conjugation ( TKC). The TKC efficiency of the wild type yeast strain was (1.2 ± 0.3) × 10^-3^

^d^ Fold increase of recipient cell number after co-cultivation (A) with or (B) without donor cells. Each value is the average of three experiments. SD = standard deviations

Differences were statistically significant compared to the wild type strain by Student’s t-test. *p <0.05, **p <0.01

### *erg28Δ* is less sensitive to growth suppression caused by co-cultivation with donor cells

**Fig. 1:**
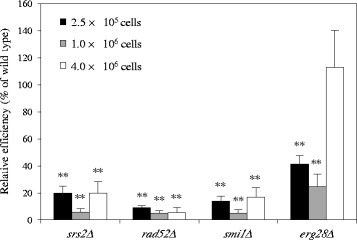
Effect of input recipient cell number on AMT efficiency. Yeast mutant strains were co-cultivated with *Agrobacterium* EHA105 harboring pBY1. The number of input yeast cells ranged from 2.5 × 10^5^ to 4 × 10^6^ cells. Relative efficiency was calculated by dividing AMT efficiency by that of yeast wild type strain. Error bars indicated the standard deviations of triplicate assays. Differences were statistically significant compared to the wild type strain to mutant strains by Student’s t-test. ** *p* <0.01

During co-cultivation with the *Agrobacterium* cells, the growth of the wild-type yeast strain was suppressed 3-fold by the presence of the donor cells (Table 1). Two mutant strains, *srs2Δ* and *smi1Δ*, showed a similar tendency to the wild-type strain. However, the growth of *erg28Δ* strain was affected very little by the presence of the donor cells (Table 1). Input recipient cell number at the start of the co-cultivation largely affected AMT with YAC type T-DNA (Table 2). We hypothesized that the continued growth of *erg28Δ* mutant cells attenuates AMT efficiency. Actually, *erg28Δ* mutant showed an AMT efficiency comparable to the wild-type in experiments that supplied a 4-fold larger number of input *erg28Δ* cells (Fig. 1). In our standard AMT experiments, 1 × 10^6^ of recipient yeast cells were added and the resulting ratio of the number of donor cells versus the number of recipient cells was 40000:1 at the start of co-cultivation (this ratio is similar to that in the mutant screening). The recipient cell number increases during co-cultivation. Even though *erg28Δ* cells grew more than the wild-type cells in the standard co-cultivation, the growth difference became negligible when the co-cultivation was started using 4-fold more yeast cells (4 × 10^6^ cells). Thus *ERG28* gene is necessary for the high efficiency of AMT but appears to be involved indirectly in the T-DNA transfer process.

**Table 2:** Effect of different recipient cell abundance on AMT efficiency

No. of input donor (cells/ml)	No. of input recipient (cells/ml)	donor : recipient input ratio	AMT efficiency^a^ (×10^-3^)	Relative efficiency (%)
4 × 10^10^	2.5 × 10^5^	160000 : 1	6.7 ± 1.7	159
4 × 10^10^	1 × 10^6^	40000 : 1	4.2 ± 0.4	(100)
4 × 10^10^	4 × 10^6^	10000 : 1	0.037 ± 0.006**	0.9

^a^ The wild type yeast cells were mixed with the donor EHA105 (pBY1) cells, and then cocultivated for 24 h. AMT efficiency is the average of three experiments

Differences were statistically significant compared to donor:recipient input ratio=40000:1 by Student’s t-test. ** *p* <0.01

### Lithium acetate transformation of the mutant strains

**Fig. 2:**
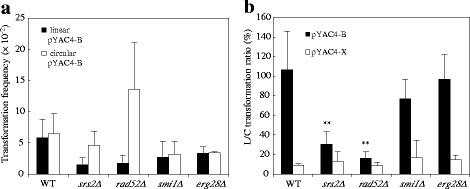
Lithium acetate transformation frequencies of yeast mutant strains. **a** Yeast cells were transformed with either 0.5 μg pYAC4-B linearized by *Bam*HI treatment or 0.5 μg of circular pYAC4-B. Transformation frequency was defined as the number of transformants per μg DNA divided by the number of viable cells after the Lithium acetate and heat-shock treatments. **b** Yeast cells were transformed with 0.3 μg of pYAC4-B or telomere-less plasmid pYAC4-X. Relative transformation frequency was expressed as the ratio of the transformation frequency obtained with linearized plasmid divided by that using circular plasmid. Error bars indicate the standard deviations of triplicate assays. Differences were statistically significant compared to the wild type strain by Student’s t-test. **p <0.01

To investigate whether the mutations identified in this screen also affect different types of transformation, we applied the lithium acetate (LiAC) transformation method. In this transformation, we used a plasmid pYAC4-B, which is derived from pYAC4. Cleavage at the *Bam*HI site adjacent to the *Tel* sequences produces linear YAC form. We used circular and linear form YACs for the transformation experiments. As shown in Fig. 2a, there was no significantly difference in the transformation frequencies between the linear and circular YAC DNA in the wild-type strain. When the transformation frequency of linear YAC DNA was normalized to that of circular YAC DNA, the L/C transformation ratio showed a value of approximately 100 % (Fig. 2b). Although the mitotic stability of short linear YACs (less than 20-kbp in size) is much lower than that of the circular ones of a similar size [23, 24], the wild-type strain was expected to maintain this linear YAC to a detectable level in this transformation. Indeed, the colonies transformed by the linear YAC showed size variation, this result might reflect the characteristics of short linear YACs, which are mitotically unstable.

The *smi1Δ* and *erg28Δ* strains exhibited average transformation frequencies that were half that of the wild-type, irrespective of whether the YAC DNA was linear or circular, but the differences were not significant (Fig. 2a), thus macromolecule uptake in general does not look seriously sick. The L/C ratio of both strains was comparable to that of wild-type strain, demonstrating that the linear YAC is also maintained in the both strains (Fig. 2b).

The *srs2Δ* and *rad52Δ* strains exhibited comparable transformation frequencies to wild type strain when using circular pYAC4-B (Fig. 2a), however these strains showed significantly low L/C transformation ratio compared with wild-type strain (Fig. 2b). This result suggested that the two genes, which are responsible for DSB repair, contribute to the stability of the linear YAC, but not the circular YAC. To confirm the stability of the YAC in the mutant strains, we also used telomere-less YAC DNA in the LiAC transformation experiments. pYAC4-X lacks any telomere sequences, and hence is expected to be predominantly maintained only as a circular form in yeast cells. In the wild-type strain, the L/C ratio with the telomere-less DNA pYAC4-X was much lower than that with pYAC4-B (Fig. 2b). This result suggested that telomere sequences of pYAC4-B are functional, and linearized pYAC4-B remains linear for a considerable time in the yeast cells. In the *srs2Δ* and *rad52Δ* strains, the mutants’ L/C frequency with pYAC4-X was the same as that of the wild-type (Fig. 2b). These results support the notion that the two genes contribute to the stability of linear YACs.

### AMT activity for integrative type T-DNA

**Fig. 3:**
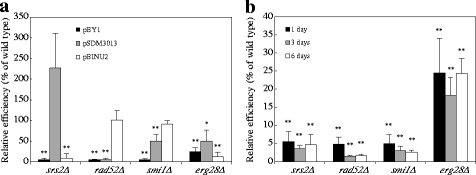
Effect of T-DNA type and co-cultivation conditions on AMT efficiency. **a** Yeast cells were co-cultivated with *Agrobacterium* EHA105 harboring a binary plasmid shown in each column. The co-cultivation time was changed depending on each donor: EHA105 (pBY1), 1 day; EHA105 (pSDM3013), 3 days; EHA105 (pBINU2), 6 days. **b** Yeast mutant cells were co-cultivated with EHA105 harboring pBY1 for the durations shown the figure. Relative efficiency was calculated by dividing the AMT efficiency by that of the wild-type strain. Error bars indicate the standard deviations of triplicate assays. Differences were statistically significant compared to the wild type strain by Student’s t-test. * *p* <0.05, ** *p* <0.01

To confirm whether the mutations would affect AMT using integrative type T-DNA, we used two *Agrobacterium* strains carrying either of two binary plasmids, pSDM3013 [2] or pBINU2. T-DNA of pSDM3013 contains the *URA3* gene flanked by *PDA1* sequences, which allow integration into the *PDA1* locus on the genome of yeast strain BY4742 through HR [2]. Because the T-DNA of pBINU2 contains only the *URA3* gene, the genome of the yeast recipient strain BY4742 has no homology with the T-DNA and thus the T-DNA integrates randomly by NHEJ. In the wild-type yeast strain, the average AMT efficiency using *Agrobacterium* strain carrying pSDM3013 was 2.5 × 10^−5^, and the efficiency using *Agrobacterium* strain carrying pBINU2 was much lower (4.0 × 10^−7^) than pSDM3013 (Table 3). In the *srs2Δ* strain, the efficiency of pSDM3013 was comparable or little higher than that of wild-type strain; however the efficiency of pBINU2 was 9 % of the wild-type strain’s efficiency (Fig. 3a). These results are consistent with the notion that mutation of the Srs2 DNA helicase causes not only a hyper-recombination phenotype [25] and high mitotic crossovers [26], but also reduction in the DSB repair process via NHEJ [27]. The *rad52Δ* mutant strain, which is deficient in the HR pathway, showed the wild-type transformation efficiency when pBINU2 was used, while the efficiency of pSDM3013 was much lower: about 5 % of the efficiency of the wild-type strain (Fig. 3a). The fact that the two mutant strains showed equal or higher efficiencies than the wild-type strain when using the integrative T-DNA suggests that Rad52 and Srs2 are involved in T-DNA modification processes after their entry into yeast nuclei, such as T-DNA integration and maintenance of replicative T-DNA.

**Table 3:** Comparison of AMT efficiency of wild type strain between replicating and integrative T-DNA

Donor strain	T-DNA features	Co-cultivation period	AMT efficiency	Relative efficiency (%/pBY1)
EHA105 (pBY1)	*Tel* ^a^ *, ARS/CEN, URA3, Tel* ^a^	1 day	(1.9 ± 0.1) × 10^-3^	(100)
EHA105 (pSDM3013)	*pda1 : : URA3* ^b^	3 days	(2.5 ± 0.4) × 10^-5^ **	1.4
EHA105 (pBINU2)	*URA3*	6 days	(4.0 ± 0.1) × 10^-7^ **	0.02
EHA105 (pBYM4)	*Tel* ^c^ *, ARS/CEN, URA3, Tel* ^c^	1 day	(9.0 ± 1.9) × 10^-3^ **	483
EHA105 (pBYM3)	*ARS/CEN, URA3*	1 day	(2.0 ± 0.3) × 10^-4^ **	11

^a^ The telomere sequence is located inside T-DNA

^b^ *URA3* gene is contained in *PDA1* locus

^c^ The telomere sequence is located in close proximity to T-DNA terminal end

Each value is the average of three experiments. Differences were statistically significant compared to EHA105 (pBY1) by Student’s t-test. ** *p* <0.01

The efficiency of the *erg28Δ* strain was consistently low with both integrative type T-DNAs (Fig. 3a), indicating that the cause of the low efficiency when using both replicating and integrative T-DNA is unrelated to maintenance or integration processes of the T-DNA. The *smi1Δ* strain restored AMT efficiency when using the two integrative type T-DNAs: its efficiencies were comparable or one half of the wild-type strain level (Fig. 3a). However, there is no report that Smi1 is involved in DNA repair pathways; thus, we speculated that changes in the co-cultivation conditions induce the restoration of AMT efficiency. In this assay, we extended the co-cultivation time with respect to each donor strain to elevate the transformation efficiency. Although we carried out the AMT experiment with different co-cultivation periods using the same donor strain EHA105 (pBY1), the *smi1Δ* strain did not restore AMT efficiency after a long co-cultivation period (Fig. 3b).

### VirE2 protein transport activity

*Agrobacterium* transfers several virulence proteins to host cells in addition to T-DNA via the T4SS. We carried out a protein transport assay to determine whether the yeast mutations also affect the transfer of the effector protein. In this assay, Cre recombinase-VirE2 fusion proteins produced in the *Agrobacterium* strain are detectable when the fusion proteins are mobilized to yeast nuclei and act on the chromosomally encoded *lox::URA3::lox* sequence. Subsequent removal of the *lox* flanked *URA3* gene caused by Cre recombination activity results in the formation of Uraˉ and 5-fluoroorotic acid (5-FOA) resistant cell [2].

**Fig. 4:**
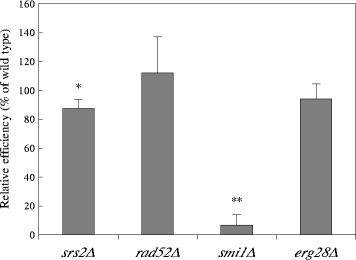
Efficiency of VirE2 protein transport from *Agrobacterium* into the yeast mutants. The yeast mutant strains that contained *lox::URA3::lox* at the *PDA1* locus were co-cultivated with *Agrobacterium* C58C1 harboring pRi1724-S3CE2 (*cre::virE2*), and then Uraˉ transformants were selected on SC medium supplemented with 0.1 % 5-fluoroorotic acid and 200 μg/ml cefotaxime. The relative efficiency was calculated by dividing by the efficiency of the wild-type strain. Error bars indicate the standard deviations of triplicate assays. Differences were statistically significant compared to the wild type strain by Student’s t-test. *p <0.05, **p <0.01

For this assay, the *lox::URA3::lox* sequence was introduced into the wild-type yeast and the mutant strains, and then the yeast cells were co-cultivated for 1 day with an *Agrobacterium* strain expressing the Cre::VirE2 fusion protein (Fig. 4). The *rad52Δ* and *erg28Δ* strains showed comparable results to the wild-type and the *srs2Δ* strain slightly reduced the efficiency (88 %). Conversely, the *smi1Δ* strain still showed a very low frequency (7 %) (Fig. 4). This result suggested that the cell-to-cell interaction, including connection of T4SS machinery between the donor and recipient cell is inhibited by *SMI1* gene deletion, at least during a short co-cultivation period, such as 1 day.

### Effect of DSB repair gene mutations on AMT

**Fig. 5:**
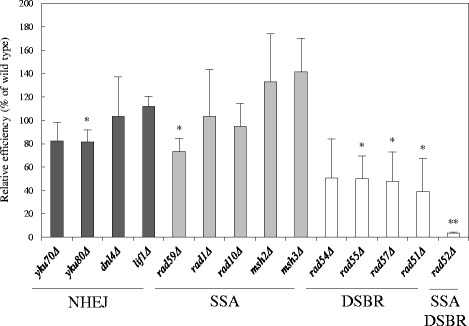
AMT efficiencies of yeast mutants defective in DSB repair by replicative T-DNA. Yeast mutant strains defective in DSB repair pathways, NHEJ, SSA and DSBR, were co-cultivated with *Agrobacterium* EHA105 (pBY1). Error bars indicate the standard deviations of triplicate assays. Differences were statistically significant compared to the wild type strain by Student’s t-test. * *p* <0.05, ** *p* <0.01

Rad52 has recombination mediator activity and promotes HR pathways, single-strand annealing (SSA) and double-strand break-repair (DSBR) [18]. In contrast, Srs2 is a DNA helicase, which acts as antirecombinase by disrupting the Rad51 presynaptic filaments [19]. The two genes obtained in the screening are involved in DNA repair pathways; therefore, we examined AMT efficiencies of mutants which are deficient in different DNA repair pathways, as shown in Fig. 5.

The AMT efficiency was not greatly affected by mutations in the genes responsible for NHEJ and SSA. The DSBR pathway defective mutations, *rad55Δ*, *rad57Δ* and *rad51Δ*, showed half the AMT efficiency of the wild-type. This result indicated that the genes involved in the DSBR pathway are important to achieve highly efficient AMT. However, the decreases caused by these mutations were not comparable with that of the *rad52Δ* strain. This result suggested that Rad52 has a function other than through the DSBR pathway that contributes to AMT. In addition, the *srs2Δ* strain has a hyper-recombination phenotype [25], meaning that frequency of DSBR is higher than in the wild-type strain. Thus, one cause of the decreased AMT efficiency in the mutant is not attributed to the deficiency of DSBR pathway: Srs2 might also have an important function that is different from the DSBR pathway and/or requires a properly regulated DSBR pathway for AMT.

### Effect of telomere-less T-DNA in the mutant strains

A study of yeast mutant strains lacking telomerase indicated that Rad52 is involved in telomere maintenance via SSA or break-induced replication (BIR) [28]. To evaluate the replication ability of transferred YAC type T-DNA further, we constructed novel binary plasmids, pBYM4 and pBYM3. The T-DNA of the binary plasmid pBY1 contains the *Tetrahymena* telomere sequences that are located at both ends of YAC, but which are within the T-DNA region. In contrast, pBYM4 has the telomere sequences close to the terminal ends (14-bp from the left border (LB) and 27-bp from the right border (RB)) of the T-DNA; pBYM3 lacks the telomere sequences at both ends. Transferred T-DNA of pBYM3 is expected to form a T-DNA circle in the recipient yeast cell. Rolloos et al*.* [13] showed that the T-DNA circles are formed in recipient yeast cells when they used some T-DNA constructs that contained *ARS/CEN* sequence but not telomere sequences.

**Fig. 6:**
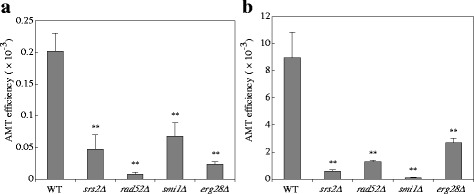
AMT efficiency of the yeast mutants by telomere-less T-DNA with and without telomere sequences. The yeast strains were co-cultivated with the donor strain **a** *Agrobacterium* EHA105 (pBYM3) or **b** EHA105 (pBYM4). Error bars indicate the standard deviations of triplicate assays. Differences were statistically significant compared to the wild type strain by Student’s t-test. ** *p* <0.01

The combination of the wild-type yeast strain and the donor with pBYM4 resulted in 4.8-fold higher AMT efficiency than that with pBY1. Conversely, the telomere-less plasmid pBYM3 reduced the efficiency to one tenth that of pBY1 (Table 3). The AMT efficiency ratio between pBYM4 and pBYM3 showed a more than 40-fold difference. These results indicated the contribution of not only the presence of telomere sequences, but also their location at the extreme ends to maintain the linear YAC type T-DNA in yeast cells. Unnecessary sequences located between the T-DNA border and the telomere sequence might interfere with the formation of a functional telomere structure at both T-DNA ends, which would explain why the T-DNA of pBY1 decreased the stability in the yeast cells compared with that of pBYM4. In each of the four mutant strains, the AMT efficiency of pBYM4 was lower than that of the wild type-strain and that using pBY1, the AMT efficiency in the mutants was also decreased when using pBYM3 (*p* <0.01) (Fig. 6a, b). These results indicated that the telomere sequence is unrelated to the low AMT efficiency of each mutant.

It is noteworthy that the results of AMT with pBYM3 were not consistent with the results of LiAC transformation using the telomere-less YAC DNA. When the linear YAC DNA lacking telomere sequences was introduced by LiAC transformation method, we could not detect significant differences in the relative transformation frequencies between the wild-type and the *srs2Δ* and *rad52Δ* mutant strains (Fig. 2b). Considering these results, we concluded that these genes play a primary role in the circularization of transferred YAC type T-DNA molecule mobilized by the T4SS, but is not required for the circularization of the YAC DNA incorporated by LiAC transformation. This difference might arise from some state of each DNA molecule entering yeast nuclei. For example, T-DNA entering into yeast nuclei is in the single-stranded form, whereas pYAC4-X molecules used in the LiAC transformation are in the double-stranded form.

### Structure of transferred DNA in the wild-type yeast strain

**Fig. 7:**
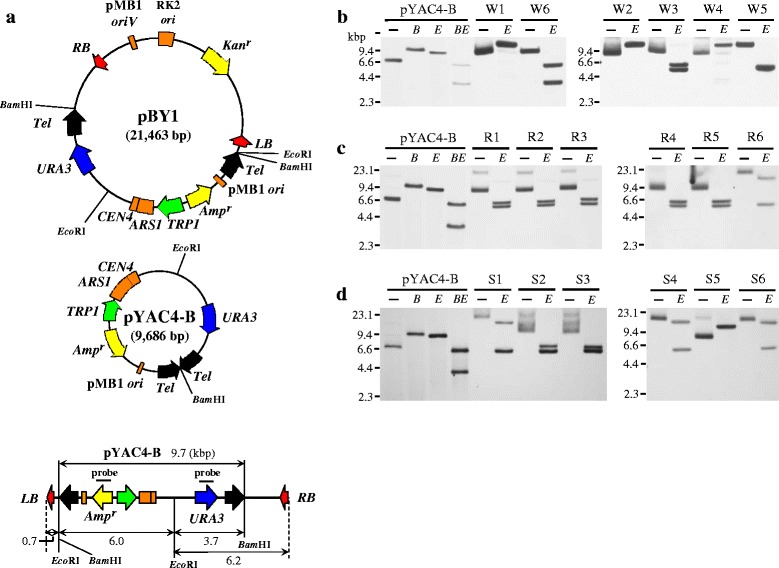
Southern blot analysis of yeast AMT transformants. AMT yeast transformants were obtained by co-cultivation with the donor bacterial strain EHA105 (pBY1). **a** Schematic diagram of the reference plasmids pBY1 and pYAC4-B, and the T-DNA region of pBY1. *Tel*, *Tetrahymena* telomere sequences. **b** Total DNA extracted AMT transformants of the wild-type yeast (W1–W6), **c** *rad52Δ* (R1–R6) and **d** *srs2Δ* (S1–S6). *Eco*RI-digested (*E*) DNA or undigested (-) DNA were used for Southern blot analysis probed with a mixture of *URA3* probe and *Amp* ^*r*^ genes (see location of each probe in panel **a**). Control DNAs were pYAC4-B (-), and pYAC4-B digested with *Bam*HI (*B*) and with *Bam*HI and *Eco*RI (*BE*)

We analyzed the T-DNA structures of six yeast transformants derived from each of the wild-type, *rad52Δ* and *srs2Δ* mutant strains by Southern blot analysis and nucleotide sequencing. Total DNA was isolated from these colonies and analysed by restriction digestion; *Eco*RI has two sites only in the T-DNA region of pBY1. One site exists in the YAC inserted in the T-DNA and another site exists outside of the YAC near the LB sequence (Fig. 7a). Subsequently, the samples were hybridized with the *URA3* and *Amp*^*r*^ probes. pYAC4-B was used as a control, *Bam*HI- or *Eco*RI-digested pYAC4-B is a 9.7-kbp linear fragment because the plasmid has a single restriction site for each enzyme. Double-digested pYAC4-B is separated into two fragments, 6.0 kbp and 3.7 kbp, respectively (Fig. 7b, c, d).

T-DNA products of two (designated W5 and W6) of the six colonies derived from the wild-type strain were linear DNA, whereas the T-DNA of the remaining four (W1–W4) were circular, based on the following results. As shown in Fig. 7b (top panel), digestion of the W6 DNA sample with *Eco*RI enzyme formed two fragments, the pattern was consistent with the double-digested pYAC4-B control. A single band was detected in the *Eco*RI-digested W5 sample, and it was suspected that two bands were overlapping (Fig. 7b). We speculated that the T-DNA was elongated by insertion or fusion of any DNA fragment into the RB side fragment after transfer to the yeast cell. Transformation of competent *E. coli* cells successfully formed Amp^r^ colonies when treated with genomic DNAs of the W1–W4 samples, whereas no Amp^r^*E. coli* colony appeared when exposed to the W5 and W6 yeast DNAs. Furthermore, PCR amplification using a set of outward-directed primers successfully produced legitimate products when the former four DNAs were used as templates DNA, while the same experiments with the latter two DNAs failed to amplify a product.

**Fig. 8:**
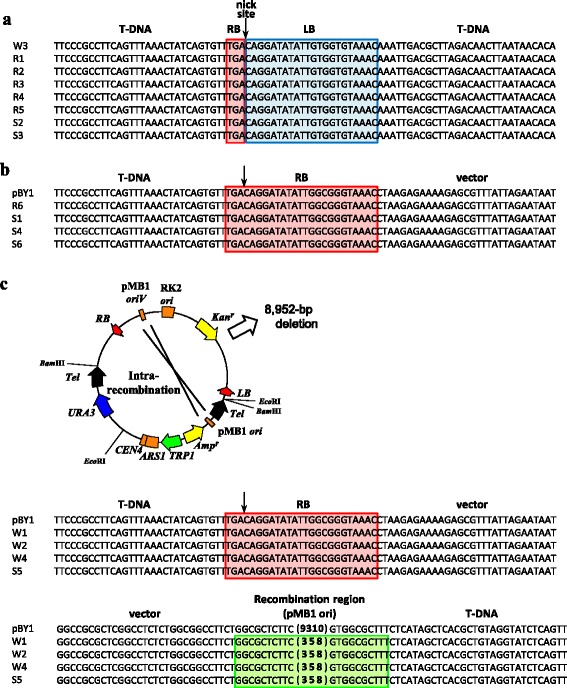
Alignment of the junction sequences in the T-DNA circles of yeast AMT transformants. DNA circles from the transformants were classified into three types. **a** T-DNA with perfect RB-LB borders junctions, **b** whole pBY1 plasmid, and **c** pBY1 with a 9310-bp deletion via intra-plasmid recombination. Nucleotide sequences derived from the RB are shaded in red, while sequences from the LB are shaded by blue. The recombination region between the two 378-bp homology sequences (pMB1 *ori*) is indicated in green, whose internal sequence is abbreviated. The schematic diagram demonstrates the recombination sites in pBY1

The restriction patterns of W1–W4 samples were classified into two types. The W3 sample digested with *Eco*RI generated approximately 6.9 and 6.0-kbp fragments, which were the approximate sizes of the fragments expected from a self-ligation of T-DNA RB and LB. Digestion of W1, W2 and W4 samples showed a single band that was shifted up compared with the uncut samples, suggesting that they comprised a circular T-DNA with a single *Eco*RI site. From the W1–W4 transformants, the DNA molecules representing T-DNA circles were rescued and their border regions were sequenced. A comparison of the sequence surrounding the T-region revealed that the W3 T-DNA circle indeed contains a junction between the transfer initiation nucleotide of RB, and the transfer end point nucleotide of LB (Fig. 8a). The T-DNA circles of the other three transformants were formed as a result of transfer of the whole pBY1 plasmid and intraplasmid recombination between a 378-bp direct repeat containing the pMB1 (ColE1/pBR322) origin. The sequences of surrounding RB region showed a perfect RB sequence connected with the vector backbone sequence and large deletion of 9-kbp by the recombination, resulting in deletion of the one *Eco*RI site (Fig. 8c).

### Structure of transferred DNA in *rad52Δ* and *srs2Δ* strains

Transferred DNAs of AMT colonies of the two repair gene mutants were also analyzed by Southern blotting and nucleotide sequencing. The results indicated that the circular DNA in AMT *rad52Δ* colonies, named R1–R5, and the AMT *srs2Δ* colonies S2 and S3 are T-DNA circles, which were the same as that of W3 (Figs. 7c, d and 8a). Similarly, we showed that the circular DNAs in AMT *rad52Δ* colony R6 and AMT *srs2Δ* colonies S1, S4 and S6 were pBY1. The whole plasmid was presumably complete pBY1 because the Southern blot profile showed two bands of 15-kbp and 6-kbp that are comparable to the plasmid, and the transfer initiation at the RB was not terminated at the LB and connected with vector backbone sequence (Figs. 7c, d and 8b). There were no DNA circles formed via intra-plasmid recombination among the AMT transformants of the *rad52Δ* strain. This observation is consistent with a mutant phenotype that is defective in HR. Rolloos et al. [13] and Bundock et al. [29] reported T-DNA circle structures being present in wild-type and *rad52Δ* strains. Our results showing the perfect border fusions and the transfer of the whole plasmid are consistent with these previous reports. The S5 transformant contained a pBY1 variant that harbors a deletion caused by intra-plasmid recombination (Figs. 7d and 8c). The two mutant strains did not contain any linear T-DNA derivatives.

## Discussion

The yeast *S. cerevisiae* has been used as a model eukaryotic recipient in experiments to study trans-domain horizontal DNA transfer phenomena. Trans-kingdom conjugation (TKC) is effected by donor bacteria harboring a wide transfer range conjugal plasmid and recipient eukaryotic cells [1]. Previously, we screened the yeast gene knock-out mutant library for strains defective in recipient ability for TKC, and found that a series of mutants lacking vacuolar ATPase activity have extremely low recipient ability [30]. In this study, we used the same set of yeast mutants to screen for low AMT ability mutants. The vATPase and other TKC-defective mutants showed a normal AMT recipient ability. In this screening, we found four low AMT-efficiency yeast mutants: *srs2Δ*, *rad52Δ*, *erg28Δ* and *smi1Δ*. By contrast, three of the four mutants are able to accept a plasmid from a donor *E. coli* at the wild-type or comparable level (Table 1) and *erg28Δ* mutant showed a quarter of the wild-type level. These data suggested that there are different limiting factors between AMT and TKC in yeast.

Smi1 protein is a regulatory protein that participates in the coordination of cell wall synthesis with bud emergence [31, 32]. To date, there has been no report that describes its contribution to DNA repair or recombination. The *smi1Δ* mutation leads to several cell wall defects, including those of bud sites, cell surface structure and its components (i.e. chitin and beta-glucan increases [22, 31, 32]). The *smi1Δ* mutant exhibited pleiotropic effects in this study. The mutant showed the low AMT efficiency with chromosomal type T-DNA and the low Cre::VirE2 accepting ability (Table 1 and Fig. 4). However, as shown in Fig. 3a, the *smi1Δ* mutation exhibited a wild-type level of efficiency for AMT with the random integration type T-DNA. We should not exclude a possibility that a small damage that give a subtle reduction in one phenomenon causes a perturbation over a labile association with the donor component and recipient cell surface target molecules. To reveal the reason why *smi1Δ* decreases the AMT efficiency when using chromosomal type T-DNA, further investigation is needed to clarify this result.

**Fig. 9:**
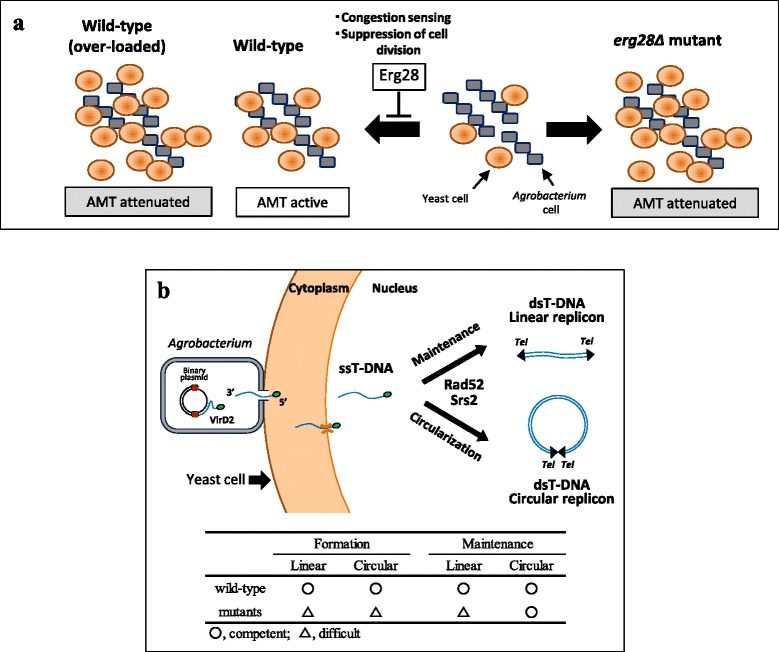
Schematic diagrams of possible roles played by *ERG28* gene and the two DNA repair genes in AMT. **a** During co-cultivation with the *Agrobacterium* cells, cell division of the wild type yeast cells is suppressed by the presence of the donor cells, while, the *erg28Δ* cells continue to grow in the same condition. *ERG28* gene product has a role in sensing congestion environment, and then suppresses cell division. Although wild-type cells can keep high AMT ability during co-cultivation, *erg28Δ* cells continue cell division and hence reduce AMT activity. When higher number of cells are loaded to co-cultivation, even wild type cells reduce AMT activity and exhibit the *erg28Δ* mutant level of low efficiency. **b** Chromosomal T-DNA contains the YAC encoding autonomous replication and segregation factors and telomere sequences. Upon the entry into nucleus, the single-stranded T-DNA (ssT-DNA) is converted into double-stranded T-DNA (dsT-DNA) and starts to replicate as a linear replicon. The *SRS2* and *RAD52* genes act soon after T-DNA entry for modification of the T-DNA to stably maintain them as linear replicons and to circularize certain T-DNA simultaneously. The linear replicon (chromosomal T-DNA) is unstable in the *srs2Δ* and *rad52Δ* mutants, hence needs circularization of the replicon, although the two mutants have lower circularization abilities and result in the formation of few AMT colonies

Erg28 is responsible for synthesis of a yeast membrane lipid component, ergosterol. Its defect causes a decrease of the lipid in cell membrane [20, 21, 33]. Erg28 protein is an endoplasmic reticulum transmembrane protein that acts as a scaffold to tether an enzymatic complex for the formation of zymosterol and also interacts with several upstream and downstream enzymes [21]. This study showed that the influence of the *erg28Δ* mutation on AMT was dependent on the cell number during co-cultivation. Cell division of the wild-type and most mutant strains was affected negatively during co-cultivation for AMT efficiency, by increasing cell number, whereas *erg28Δ* mutant cell division was unaffected by the presence of donor bacterial cells as shown in Table 1. When provided with a large quantity of cells at the beginning, congestion in the co-cultivation suppressed not only AMT, but also cell division of wild-type cells in general. The *erg28Δ* mutant cells continued to grow despite the presence of the donor bacterial cells and the congestion (Table 1), and thereby attenuated AMT efficiency much more than the wild type strain (Fig. 9a). Actually, the *erg28Δ* mutant showed an AMT efficiency comparable with the wild-type strain in the experiments that supplied a larger number of recipient cells (Fig. 1). Thus, this gene is not directly involved in T-DNA transfer process, but is required for high efficiency AMT.

This study indicated that AMT is affected seriously not only by *rad52Δ*, but also by *srs2Δ*. The lack of the *SRS2* gene is as deleterious in AMT as the lack of *RAD52*, even though *srs2* mutations enhance HR repair [18, 19, 25, 26]**.** In fact, the *srs2Δ* mutant showed high AMT efficiency in AMT with the homologous integration type T-DNA. It is likely that a special role in strand exchange by Rad52 and a controlled (unbiased) action among repair genes are important in AMT. The importance of the HR repair gene *RAD52* in AMT was already shown by van Attikum et al*.* [12] for the transfer of T-DNA having sequences identical with yeast chromosomal genes, and by Rolloos et al*.* [13] for the transfer of T-DNA containing autonomously replicating sequences. In contrast, the other mutants defective in DSBR through HR, such as the mutant of the *recA* homolog gene *RAD51,* exhibited only 50 % defect in AMT, as shown in Fig. 5.

We examined the mutants based on their transformability by the LiAC method, using circular and linear forms of YAC DNAs. Both types of DNA resulted in similar transformation frequencies in the wild-type strain. The repair mutants *rad52Δ* and *srs2Δ* supplied with linearized YAC DNA exhibited apparently lower frequency than with circular YAC DNA (Fig. 2a). The linear YAC DNA via LiAC transformation and the T-DNA via AMT can either keep the linearity or form a circular molecule in wild type recipient cells as schematically shown in Fig. 9b. Previous studies reported lower mitotic stability of linear artificial chromosome DNAs in yeast when the DNA length is around 10-kbp [23, 24]. Partitioning of the small chromosomes into daughter cells is attenuated according to the literature [23, 24]. Indeed, in this study the wild-type strain given linearized YAC DNA produced various sizes of transformed colonies, while the strain provided with the circular YAC DNA produced even sized colonies (data not shown). However, there was only a small difference in the transformation frequency of the wild-type strain between the linearized and circular YAC DNAs. The lower frequency of the two mutants suggested lower mitotic stability, hence the need for circularization of the linearized YAC DNA for stabilization, although the two mutations have lower circularization abilities and result in the formation of few transformed colonies. Accordingly, every colony of the two mutants contained circularized YAC type T-DNAs, as shown in Fig. 7c and d. The lower AMT efficiencies in the *rad52Δ* and *srs2Δ* mutants suggested that an appropriate level of HR is important for T-circle formation (Fig. 6a, b). Taken together, the results suggested that the linear YACs were unstable in the two mutants, and thereby AMT transformants retained circularized YACs, which are stable, even in the mutants. Paradoxically, however, the T-circle formation is stuck by the mutations. Therefore, *rad52Δ* and *srs2Δ* mutants cause severe defect in the AMT with pBY1. We concluded that these genes are required for the final step of the T-DNA transfer process, such as integration into the recipient genome and the formation of T-circles. Both processes are important in the stable maintenance of the received integrative or replicative type T-DNAs.

Rolloos et al*.* [13] proposed a model for T-circle formation. According to the model, Rad52 promotes mobility of T-DNA terminals and helps ligation between the terminals by the strand transferase activity of VirD2 and hence concatemer formation. The concatemers are then converted into smaller circles through HR. Our analysis of the T-DNA derivatives of pBY1 indicated that T-DNA is circularized in every AMT transformant colony examined: the circularized molecules were monomers and no concatemers formed from T-circles were found. The result is reasonable, because, in general, ligation between the two ends of a single T-DNA molecule might take place at a higher probability than that between different molecules at a low DNA concentration. It needs to be considered that pBY1 contains a centromere and therefore concatemer formation inevitably causes the problem of a multi-centric molecule. Dicentric molecules are unstable because of their inappropriate distribution during mitotic cell division [34]. The same authors mentioned that dicentric molecules are still maintainable. The mechanism of how the HR pathway is involved in T-circle formation and the eventual linkage between the two ends formed, remains to be revealed.

In papers dealing with T-DNA integrated in chromosomes and T-circles, T-DNA repeats were often observed, and the repeats showed every type of topology between the molecules, i.e. RB-LB, RB-RB and LB-LB junctions [7, 35–38]. The authors suggested that replication of a transferred, single-stranded T-DNA precedes the concatenation reaction, based on the fact that the topology is not unique but variable. Partial deletions in T-DNA with very high frequency deletions in the LB side were described in several articles [7, 39, 40]**.** Liang & Tzfira [41] explained that the deletions take place at the replication step. Their notions support the view that double-stranded T-DNA is the substrate for integration and circularization events [7]. In contrast to the T-DNA found in plants, no inverted repeat with RB-RB or LB-LB junctions was observed in yeast [13]. Similarly, the circles shown in Fig. 8 have very few or no deletions. We speculated that the transferred, single-stranded T-DNA forms a circle in yeast cells upon the entry from the donor *Agrobacterium* cells. The possible reaction at the early single-stranded form stage gives advantage to the linkage between its 5′ and 3′ ends and minimizes deletion. This hypothesis correlates with the characteristics of Rad52 and Rad51 proteins in that they associate with single-stranded DNA [18]. By contrast, T-DNAs integrated in yeast chromosomes exhibited deletions at the LB side boundary [11]. The latter T-DNAs might be integrated after the replication reaction, like those in plants.

The different T-DNA structures between the yeast and plant cells could be explained if we assumed that the terminal end ligation and circularization events occurs at different times between the yeast and plant cells. This might reflect the difference in major DNA repair pathways between yeast and plants. In plants, NHEJ is the major repair pathway [42], where NHEJ is carried out between double-stranded DNA terminals, and thereafter, T-DNA replication precedes integration into chromosomal DNA. In yeast, the HR repair pathway is most active [43] where Rad51 and Rad52 interact with single-stranded DNAs. We assume that the HR factors bind to and protect newly received T-DNA, and promote the association between their two ends and circularization.

## Conclusion

Contribution of not only *RAD52* but also the DNA helicase/antirecombinase gene *SRS2* is necessary for the linear artificial chromosome formation and maintenance as well as for AMT efficiency through the transfer. A sterol synthesis scaffold gene *ERG28* is important in high-efficiency AMT, possibly by avoiding congestion. Necessity of secured cell surface is confirmed as a prerequisite by the effect of the cell wall synthesis regulator *SMI1*. These data and resources made in this study would benefit further study in molecular level and development of new vectors.

## Methods

### Microbial strains and culture conditions

Bacterial and yeast strains used in this study are listed in Table 4. Yeast strains BY4742 and the complete set of *MATα* knockout mutants (the *Saccharomyces* Genome Deletion Project consortium) derived from BY4742 were purchased from Invitrogen (Carlsbad, CA, USA) Bacterial strains and yeast strains were cultured as described previously [16]. Transformation of yeast strain BY4742 and its derivative strains were routinely selected on SC medium lacking uracil (SC-ura; 2 % glucose, 0.67 % Difco yeast nitrogen base without amino acids, 0.003 % lysine, 0.002 % histidine and 0.003 % leucine).

**Table 4:** Bacterial and yeast strains, and plasmids used in this study

Strain or plasmid	Relevant genotype and/or characteristics	Reference or source
*Agrobacterium tumefaciens* strains		
C58C1	Ti plasmid-less C58rif; Rif^r^	Our collection
EHA105	C58 containing pTiEHA105 (T-DNA deletion)	[50]
*Escherichia coli* strain		
HB101	*F* ^*-*^, *recA13*, *proA2*	[51]
SURE	F′[lacIq lacZΔM15] lac recB recJ sbcC umuC::Tn5(KanR) uvrC	Stratagene
*Saccharomyces cerevisiae* strains		
BY4742	*MATα his3Δ1 leu2Δ0 lys2Δ0 ura3Δ0*	[52]
Mutants derived from BY4742		The yeast genome deletion project
BY4742floxU	BY4742 with *loxP::URA3::loxP* inserted in *pda1*	This study
BY4742 mutants with *loxP::URA3::loxP*		This study
Plasmids		
pBIN19	Binary vector with an artifical T-DNA (*LB*, *P*_*nos*_:*nptII*, *lacZ’* with MCS, *RB*) and *nptIII*; Km^r^	[44]
pYAC4	Yeast artificial chromosome; *HIS3,* (*Tel*, *TRP1*, *ARS1*, *CEN4*, *URA3*, *Tel*); Amp^r^ (Car^r^)	[45]
pYAC4-B	*Bam*HI-digested and ligated pYAC4; (*Tel*, *TRP1*, *ARS1*, *CEN4*, *URA3*, *Tel*); Amp^r^ (Car^r^)	This study
pYAC4-X	*Xho*I-digested and ligated pYAC4; (*TRP1*, *ARS1*, *CEN4*, *URA3*); Amp^r^ (Car^r^)	This study
pBY1	pBIN19 containing (*Tel*, *TRP1*, *ARS1*, *CEN4*, *URA3*, *Tel*) at MCS in T-DNA; Km^r^ and Amp^r^ (Car^r^)	[16]
pBIN19Δ	pBIN19 having nothing but 33 nucleotides including *Bam*HI cutting site between *LB* and *RB* A PCR product.	This study
pBINU1	pBIN19Δ harboring *URA3* at *Bam*HI site in T-DNA; Km^r^	This study
pBINU2	pBINU1 lacking the 52-bp homology segment	This study
pBYM3	pBIN19Δ harboring (*TRP1*, *ARS1*, *CEN4*, *URA3*) at *Bam*HI site in T-DNA; Km^r^ and Amp^r^ (Car^r^)	This study
pBYM4	pBIN19Δ harboring (*Tel*, *TRP1*, *ARS1*, *CEN4*, *URA3*, *Tel*) at *Bam*HI site in T-DNA; Km^r^ and Amp^r^ (Car^r^)	This study
pSDM3013	pBIN19 with *pda1::loxP::URA3::loxP::pda1*at MCS in T-DNA; Km^r^	[2]
pRi1724-S3CE2	pRi1724-S3 containing *P*_virE_ *::virE1::cre::virE2* fusion in place of a region starting from *riorf135* to *riorf144*; Gm^r^	[16]
pRS313	*HIS3*, *ARSH4/CEN6* and *Amp* ^r^ (*Car* ^r^)	[53]
pSRS2	*SRS2* in pRS313	This study
pRAD52	*RAD52* in pRS313	This study
pSMI1	*SMI1* in pRS313	This study
pERG28	*ERG28* in pRS313	This study
pRH210	*oriV*, *oriT* ^*incP*^, *mob* ^*incP*^, *tra* ^*incP*^ and *Amp* ^r^	[54]
pAY205	*ARS1*, *TRP1*, *URA3*, *oriV* ^*incQ*^, *oriT* ^*incQ*^, *mob* ^*incQ*^ and *Km* ^r^	[54]

### Plasmid construction

The plasmids used in this study are listed in Table 4 and the primers used in this study are listed in Table 5. For the complementation experiments, each wild-type yeast chromosomal gene was amplified by PCR using a set of primers designed based on the genomic sequence: one 0.5-kbp upstream and another 0.5-kbp downstream of the target ORF. As shown in Table 5, recognition sequences for restriction enzymes were added to the 5′ part of the primer sequences, where necessary. Each of the amplified chromosomal genes was cloned into pRS313.

**Table 5:** Oligonucleotide primers used in this study

Primer	Resultant construct	Sequence (5′-3′)
BamINLB	pBYM3, pBYM4 and pBINU1	CGGGATCCTCAATTTGTTTACACCAC
BamINRB	pBYM3, pBYM4 and pBINU1	CGGGATCCCAGTTTAAACTATCAGTG
SRS2Fw	pSRS2	GGAATTCCAGGAGTGAAGACATCTGC
SRS2Rv	pSRS2	GGAATTCCGACTTGGGACTATTGGAC
RAD52Fw	pRAD52	GGAATTCTAACGGTGAGTGTGGCAACG
RAD52Rv	pRAD52	GGAATTCTGAACCTAAGGATTCCGCTG
SMI1Fw	pSMI1	GGAATTCCACTGATCCATTTACCTGC
SMI1Rv	pSMI1	GGAATTCCTCATGCTCTTCAATGTCG
ERG28_2_Fw	pERG28	CGAGGAATTCTCGGGGACAACAACTTCAG
ERG28Rv	pERG28	GGAATTCTGAGCTGGAGCAGACATTG
URA3-BamHIfw	pBINU1and pBINU2	CGGGATCCCGAGTCGCATAAGGGAGAGC
URA3-BamHIrv	pBINU1	CGGGATCCCGATCAGCGTGGTCGTGAAG
URA3-EcoRI	pBINU2	CGGAATTCCTGAAGCTCTAATTTGTG
URA3-probe-Fw	*URA3* probe	CTTAACCCAACTGCACAGAACA
URA3-probe-Rv	*URA3* probe	GCAATAAAGCCGATAACA
Amp-probe-Fw	*Amp* ^r^ gene probe	TGCAATGATACCGCGAGAC
Amp-probe-Rv	*Amp* ^r^ gene probe	CGAACTGGATCTCAACAGCGGTAA
pBY1-RB-Seq	for sequencing	GAACGCGCAATAATGGTTTCTG
pBY1-RB-Seq2	for sequencing	GGGCGCACCGCAGATGGAAA

Binary plasmids pBYM4 and pBYM3 were constructed as follows. An 8.6-kbp DNA fragment was amplified using primers BamINLB and BamINRB using pBIN19 [44] as the template. The resulting 8.6-kbp T-DNA-less PCR product was digested with *Bam*HI (pBIN9Δ), and then ligated into pYAC4 [45] and cleaved with the same enzyme, resulting in an 18.3-kbp plasmid pBYM4. Similarly, pYAC4 was digested with *Xho*I, and its cohesive ends were filled in using Klenow fragment (Nippon gene, Tokyo, Japan). The fragment was ligated with the 8.6-kbp pBIN19 PCR product, resulting in a 17.9-kbp plasmid pBYM3.

A binary plasmid pBINU1 was constructed by the ligation of two fragments. pBIN19 was amplified using the BamINLB and BamINRB primers and the *URA3* gene was amplified using URA3-BamHIfw and URA3-BamHIrv primers from yeast genomic DNA of a descendant of the S288C strain KS13-1D [46]. The two PCR products were digested with *Bam*HI, and ligated to form plasmid pBINU1. This plasmid contains 52 bp of homology with the yeast BY4742 genomic DNA. Plasmid pBINU2 is a derivative of pBINU1 that lacks the 52-bp region. pBINU1 was used as a template for PCR using the primers URA3-BamHIfw and URA3-EcoRI. The resulting PCR product was blunt-ended, and then self-ligated to produce pBINU2.

Plasmid pYAC4-B and pYAC4-X were constructed by self-ligation of *Bam*HI- or *Xho*I-digested pYAC4.

### Initial screening of AMT-defective yeast mutants

For mutant screening using the yeast deletion strains, the following co-cultivation and selection was carried out. *Agrobacterium* cells harboring the binary plasmid pBY1 [16] were grown overnight in liquid LB media supplemented with appropriate antibiotics. The bacterial cells were collected, resuspended in liquid AB induction medium [16] at an OD_660_ of 0.5, and then incubated at 28 °C for 15-18 h. Yeast strains were stamped on YPD agar using pin replicator and incubated overnight at 28 °C for preculture. A 5 μl sample of the cell suspension (2 × 10^10^ cells/ml) of the donor *Agrobacterium* strain was spotted on solid AB induction medium supplemented with 50 μg/ml carbenicillin, the required amino acids and uracil. A small quantity of yeast cells was taken from the overnight YPD agar culture, and then mixed with the donor *Agrobacterium* cell suspension on the solid AB induction medium using a plastic inoculation loop. The mixture was kept for 24 h at 22 °C, and then transferred using a plastic inoculation loop onto SC-ura agar containing 200 μg/ml cefotaxime, which is selective for the resulting yeast transformant cells. The proportions of transformant colonies of each strain were confirmed visually. To confirm whether the recipient yeast strains exhibited auxotrophy other than uracil, the yeast strains were also transformed using eutrophic media (solid AB induction medium and SC-ura agar supplemented with 0.5 % casamino acid, 0.002 % tryptophan and 0.002 % adenine).

### AMT efficiency test

AMT efficiency was determined as described by Bundock et al. [29] and Kiyokawa et al. [16], with the following modifications. Donor *Agrobacterium* cells were prepared as above. Recipient yeast strains were grown overnight in liquid YPD medium at 28 °C.A 10 μl sample of the cell suspension of the donor bacterium and a 10 μl sample of the cell suspension (1.3 or 2.5 × 10^6^ cells/ml) of the each of yeast strain were mixed and then spotted onto solid AB induction medium. Donor cells harboring YAC type T-DNA were co-cultivated for 24 h at 22 °C, whereas the co-incubation duration was extended to 3–6 days for integration type T-DNA transfer. After co-cultivation, the *Agrobacterium*–yeast cells mixture was resuspended and then spread onto a solid SC-ura agar containing 200 μg/ml cefotaxime. Yeast AMT efficiency was calculated by dividing the AMT transformant colony number by the output yeast cell number.

Quantitative TKC efficiency test was carried out essentially as described by Mizuta et al. [30].

### Yeast transformation by the lithium acetate transformation method

Yeast transformation by the LiAC transformation method was performed as described by Gietz et al. [47]. pYAC4-B and pYAC4-X were digested or not with *Bam*HI or *Xho*I to obtain circular and linear DNA fragments. Samples of 0.3 or 0.5 μg of these DNA were used for transformation. The transformation frequency was defined as the number of transformants (per μg DNA) per viable cell number. Relative transformation frequencies for each genotype were expressed as the ratio (%) of transformation frequency obtained with linear DNA divided by that of circular DNA.

### Protein transport assay

Transport of cre recombinase and its fusion proteins was assayed as described previously [16], with some modifications. This assay was similar to the method used to determine AMT efficiency in the mutant screening until the co-cultivation step. After co-cultivation, the cell mixture was resuspended and spread onto solid SD medium supplemented with lysine, histidine, leucine, uracil, 0.1 % 5-fluoroorotic acid (5-FOA), and 200 μg/ml cefotaxime. After 3 days of incubation, 5-FOA-resistant colonies were counted. The efficiency of *URA3* gene excision was calculated by dividing the 5-FOA-resistant colony number by the output yeast cell number.

### Southern blotting and sequence analysis

Transformed yeast cells were grown in 100 ml of SC-ura and DNA was extracted using the method described by Devenish & Newlon [48]. The purified DNA (0.5 μg) from each yeast transformant and pYAC4-B (2 ng) were digested with or without *Eco*RI and separated by electrophoresis through a 0.8 % agarose gel. Southern blot transfer to positively charged Nylon membrane was performed by a standard neutral capillary transfer method. The membrane was probed with *URA3* and Ampicillin resistance gene probes. 600-bp *URA3* gene fragment and 601-bp *Amp*^*r*^ fragment, respectively, were produced by PCR amplification using primers URA3-probe-Fw and URA3-probe-Rv, or Amp-probe-Fw and Amp-probe-Rv. The probe preparation, hybridization and detection were carried out using the AlkPhos Direct Labelling and Detection System (GE Healthcare, Little Chalfont, England), according to the manufacturer’s protocol.

Circular form T-DNA in yeast cells was recovered into *E. coli* cells according to the method of Inoue et al. [49]. HB101 or SURE *E. coli* transformant colonies were selected on LB medium containing ampicillin (50 μg/ml). Circular T-DNA was extracted from each transformant and sequenced using the BigDye® Terminator v3.1 Cycle Sequencing Kit (Applied Biosystems, Foster, CA, USA), following the protocol provided by the manufacturer. A primer, pBY1-RB-Seq, anneals just inside the RB sequence, and primer pBY1-RB-Seq2 is near the pMB1 origin sequence of pBIN19 vector backbone.

### Statistical analysis

All data shown are representative of at least 3 independent experiments and represented as mean of the performed experiments with standard deviation. Statistic tests were done with two-tailed Student’s *t*-test. Statistical analysis was performed using a function equipped in Microsoft Excel 2010.
